# Construction of Immobilized Laccase System Based on ZnO and Degradation of Mesotrione

**DOI:** 10.3390/toxics12060434

**Published:** 2024-06-16

**Authors:** Wanlei Yue, Xin Wang, Jiale Zhang, Jia Bao, Mengqin Yao

**Affiliations:** 1 School of Environmental and Chemical Engineering, Shenyang University of Technology, Shenyang 110870, China; m15222487370@163.com (W.Y.); 18704120001@139.com (J.Z.); baojia@sut.edu.cn (J.B.); 2 School of Chemistry and Chemical Engineering, Guizhou University, Guiyang 550025, China; mqyao@gzu.edu.cn

**Keywords:** immobilized laccase, mesotrione degradation, ZnO nanomaterials

## Abstract

Mesotrione (MES) is a new environmental pollutant. Some reports have indicated that microbial enzymes could be utilized for MES degradation. Laccase is a green biocatalyst whose potential use in environmental pollutant detoxification has been considered limited due to its poor stability and reusability. However, these issues may be addressed using enzyme immobilization. In the present study, we sought to optimize conditions for laccase immobilization, to analyze and characterize the characteristics of the immobilized laccase, and to compare its enzymatic properties to those of free laccase. In addition, we studied the ability of laccase to degrade MES, and analyzed the metabolic pathway of MES degradation by immobilized laccase. The results demonstrated that granular zinc oxide material (G-ZnO) was successfully used as the carrier for immobilization. G-ZnO@Lac demonstrated the highest recovery of enzyme activity and exhibited significantly improved stability compared with free laccase. Storage stability was also significantly improved, with the relative enzyme activity of G-ZnO@Lac remaining at about 54% after 28 days of storage (compared with only 12% for free laccase). The optimal conditions for the degradation of MES by G-ZnO@Lac were found to be 10 mg, 6 h, 30 °C, and pH 4; under these conditions, a degradation rate of 73.25% was attained. The findings of this study provide a theoretical reference for the laccase treatment of 4-hy-droxyphenylpyruvate dioxygenase (HPPD)-inhibiting herbicide contamination.

## 1. Introduction

Mesotrione (MES) is a commonly used herbicide that inhibits 4-hydroxyphenylpyruvate dioxygenase (HPPD), which affects the production of carotenoids and photosynthesis in plants [1]. MES is weakly acidic with a pKa of 3.12 at 20 °C; it is also stable, not easy to volatilize, soluble in water, and can also be well dissolved in organic solvents [2]. It is a highly efficient triketone herbicide widely used for controlling broadleaf and grassy weeds in maize, sugarcane, and rice cultures [3,4]. However, after MES is applied in the field, it may remain in the soil and on the surfaces of plants, subsequently penetrating soil and water and causing harm to the environment. A number of studies have shown that MES has significant inhibitory effects on the survival of bee colonies [5] and on the growth of Vallisneria natans [6]. The treatment of MES residues in the environment is, therefore, a necessary task.

The degradation of MES is typically achieved through physical and chemical methods, in addition to biodegradation. For example, high-temperature plasmas [7] and the electro-Fenton process [8] can be used to degrade MES. However, these methods require additional catalysts, and their associated costs are relatively high. In comparison, the use of biodegradation involves no toxicity and results in no secondary pollution. The degradation of HPPD herbicides (such as MES) has been shown to be feasible in many experimental studies [9,10,11]. Laccase in particular has shown potential application value in the degradation of herbicides [9,12,13]. Laccase (EC 1.10.3.2) is a copper-containing polyphenol oxidase which exhibits high catalytic efficiency [14]. The catalytic sites for laccase are typically four copper atoms, that is, T1, T2, and two T3s [15]. T1-Cu, having the highest redox potential, serves as the oxidation site of the substrate, and is linked to two histidines and one cysteine as a ligand; T2 and two T3 Cu atoms combine to form trinuclear copper cluster (TNC), linking to histidine as a side chain. Consequently, laccase transfers an electron from the pollutant to TNC via a His-Cys-His tripeptide and converts O_2_ to H_2_O in collaboration with aspartic and glutamic acids. Two carboxylic acid groups from the aspartic and glutamic acids provide hydrogen atoms for reducing O_2_ to H_2_O [14,16,17]. In addition, because of its excellent catalytic oxidation ability, laccase is widely used in organic synthesis [18], food processing [19], textile-dye decolorization [20,21], environmental-pollutant detoxification [22], and biosensors [23]. However, the application of free laccase in pollutant degradation involves certain limitations relating to stability, reusability, and activity retention across wide ranges of temperatures and pH values. Addressing these limitations is essential if laccase is to be implemented on a large scale, and immobilization of laccase represents one potential means of doing so [17]. Yang et al. [24] co-immobilized laccase using MOFs and hydrogels as carriers, and found that the immobilized laccase exhibited excellent stability. Mohie E.M. Zayed et al. [25] immobilized laccase with poly(methylmethacrylate) (PMMA) composites, they found that the immobilized laccase had a wider temperature range and exhibited a 4.6-fold improvement in storage stability. The carrier is one factor that affects the immobilization effect of enzymes. Metal nanomaterials are characterized by high specific surface areas and excellent thermal and mechanical stabilities, and are extensively used in the field of enzyme immobilization [26]. Bruera et al. [27] immobilized laccase on nanoporous alumina for black liquor treatment; they found that immobilized laccase exhibited better pH stability and removal efficiency than free laccase. Because of their excellent biocompatibility, chemical stability, and non-toxicity, ZnO nanomaterials have been widely used as chemical sensors and antibacterial agents, as well as for environmental applications [28,29,30]. Jantiya Isanapong et al. [31] evaluated the efficiency of immobilized laccase for the degradation of tertiary butyl alcohol (TBA) on ZnO nanostructures; they found that immobilized laccase exhibited better stability and degradation. Manviri Rani et al. [32] prepared lac-ZnO nanospheres and found that they exhibited improved enzymatic properties compared with free laccase. In addition, ZnO nanomaterials have excellent adsorption properties with respect to organic and inorganic pollutants in the water matrix [33].

For the present study, we carried out research concerning the preparation and screening of nano-ZnO carriers with different morphologies. We then constructed an immobilized laccase system, which was used for the degradation of MES, after optimization of conditions and discussion of enzymatic properties.

## 2. Materials and Methods

### 2.1. Materials

Laccase (EC 1.10.3.2) was purchased from Novozymes (China) Biotechnology Co., Ltd. (Tianjin, China). 2,2-azinobis-3-ethylbenzothiazoline-6-sulfonate (ABTS) was purchased from Kuer Chemical Technology Co., Ltd. (Beijing, China). Mesotrione ((2(4-methylsulfonyl)-2-nitrobenzoyl) cyclohexane-1,3-dione, purity > 97%) was purchased from Guangzhou Puxin Biotechnology Co., Ltd. (Guangzhou, China).

### 2.2. Preparation and Characterization of ZnO Nanomaterials

ZnO nanomaterials were prepared using the hydrothermal reaction method. Ethylene glycol (EG) and deionized water (190 mL in total volume) were added to a beaker in measured proportions (2:1, 1:1, 1:3, 1:4, or 1:6), and then stirred and mixed for 5 min. Zn(COOH)_2_·2H_2_O (1.317 g) was dissolved in the mixture until the mixture became clear. NaOH solution (3.6 M, 10 mL) was then added to the mixture and stirred for 30 min. The obtained mixture was transferred into a polytetrafluoroethylene (PTFE) reactor (Shanghai Jinghong Experimental Equipment Co., Ltd., Shanghai, China) and allowed to stand at 140 °C for 12 h. After centrifugal separation of the product, the obtained precipitate was washed with ethanol and deionized water, then dried at 100 °C for 3 h to obtain powdered ZnO. Scanning electron microscopy (SEM, Carl Zeiss AG, Oberkochen, BW, Germany) was used for microscopic morphology analysis of the ZnO product. The prepared ZnO was then characterized by Fourier transform infrared spectroscopy (FT-IR, Thermo Nicolet Corporation, Madison, WI, USA).

### 2.3. Determination of Laccase Activity

Laccase solution (0.5 mg/mL, 0.2 mL) was added to a reaction system consisting of ABTS solution (0.5 mM, 1.9 mL) and disodium hydrogen phosphate–citric acid buffer solution (pH 4, 1.9 mL) at 60 °C for 3 min. Absorbance was measured using an ultraviolet spectrophotometer (UV, Shanghai Precision Scientific Instrument Co., Ltd., Shanghai, China) at 420 nm before and after the reaction. The methodology for determining immobilized laccase activity was identical to that described above. Enzyme activity was calculated using Equations (1) and (2) as follows:

(1) $$\mathrm{Unit}\mathrm{enzyme}\mathrm{activity}\mathrm{of}\mathrm{enzyme}\mathrm{solution}\left(U\right)=\frac{10^{6}\times \Delta \mathrm{OD}\times V_{1}}{V_{2}\times \varepsilon \times \Delta t}$$

(2) $$\mathrm{Immobilized}\mathrm{laccase}\mathrm{unit}\mathrm{enzyme}\mathrm{activity}\left(U\right)=\frac{10^{6}\times \Delta \mathrm{OD}}{\varepsilon \times \Delta t\times M}$$

where V_1_ is the total volume of the reaction system (4 mL); V_2_ is the volume of enzyme solution added to the system (0.2 mL); ΔOD is the change in absorbance values within time Δt; Δt is the reaction time (3 min); M is the mass of immobilized laccase (g); and ε is the molar extinction coefficient of the substrate ABTS at 420 nm, with a value of 3600 L·μmol^−1^·cm^−1^.

### 2.4. Relative Enzyme Activity and Enzyme Recovery Rate

Relative enzyme activity refers to a calculation for which the group with the highest enzyme activity was given a value of 100%; the levels of enzyme activity measured in other groups were then compared with this, giving values for relative enzyme activity. The rate of enzyme activity recovery was calculated using Equation (3), as follows:

(3) $$\mathrm{enzyme}\mathrm{activity}\mathrm{recovery}\mathrm{rat}=\frac{A_{1}}{A_{2}}\times 100\%$$

where A_1_ is the initial total enzyme activity of the enzyme solution; and A_2_ is the total immobilized enzyme activity.

### 2.5. Determination of Laccase Immobilization Capacity

Bradford reagent (5 mL) was added to the pre-immobilized laccase solution and the post-immobilized supernatant (1 mL each). The solution was shaken well and allowed to stand for 5 min before measurement. Absorbance was measured at 595 nm, and the laccase fixation amount was calculated using Equation (4), as follows:

(4) $$q=\frac{{(C}_{0}-C)\times V}{m}$$

where q is the fixed capacity of the immobilized material for laccase (mg/g); C_0_ is the initial concentration of laccase solution (mg/g); C is the concentration of laccase in the supernatant at fixed equilibrium (mg/g); V is the volume of the fixed solution (mL); and m is the mass of the immobilized material (mg).

### 2.6. Preparation of Immobilized Laccase

ZnO nanomaterial (10 mg) was added to laccase solution (20 mL, 0.5 mg/mL) which prepared by dissolution in a buffer solution (pH 7), and cultured in a constant temperature shaker (150 rpm). The immobilized laccase was filtered and separated after a certain time. To determine the best immobilization conditions, five immobilized materials (S-ZnO, C-ZnO, G-ZnO, F-A-ZnO, and F-B-ZnO) were studied using orthogonal experiments at temperatures of 20, 30, 40, 50, and 60 °C, adsorption times of 0.5, 1, 2, 3, and 4 h, and enzyme concentrations of 5, 10, 15, 20, and 25 mg/mL.

### 2.7. Determination of Kinetic Constants

According to the method described in Section 2.3 above, at the optimal temperature and pH values for free and immobilized laccase, respectively, the concentration of ABTS solution was set to 0.02, 0.04, 0.06, 0.08, and 0.10 mM, successively, for the purposes of analysis. To this end, the Michaelis Equation was used, as in Equation (5).

However, because the above is limited in application, the Lineweaver–Burk double reciprocal method is also commonly used, as illustrated in Equation (6), as follows:

(5) $$v=\frac{V_{max}\times S}{K_{m}+S}$$

(6) $$\frac{1}{v}=\frac{K_{m}}{V_{max}}\times \frac{1}{S}+\frac{1}{V_{max}}$$

where v is the initial rate of enzymatic reaction; S is the concentration of substrate ABTS; V_max_ is the maximum reaction rate; and K_m_ is the Michaelis constant.

### 2.8. Detection and Extraction of MES

The reaction substrate in this experiment was 10 mg/L aqueous solution of MES. The concentration of MES was determined during the analysis using high-performance liquid chromatography (HPLC), and the HPLC detection method was employed for MES with an external standard method using Agilent 1260 high-performance liquid chromatography (Agilent Technologies Inc., Shanghai, China). The mobile phase consisted of a solution of 0.7% glacial acetic acid in aqueous solution and acetonitrile. The column temperature was set to 25 °C, the detection wavelength was 252 nm, the flow rate was 1.0 mL/min, and the injection volume was 20 μL.

The solution containing MES (5 mL) and acetonitrile (5 mL) was mixed and shaken on a shaker (180 rpm/min, 30 °C) for 30 min. NaCl (1 g) was then added and the mix was centrifuged at 4000 rpm for 5 min. The upper layer of organic liquid filtration (0.22 μm filter membrane) was taken and refrigerated at −20 °C for testing. The recovery rate of MES in an aqueous solution was then calculated to be 83.3%, indicating that the method satisfied the requirements of the experiment.

### 2.9. Data Analysis

All experimental treatments were repeated three times. Charts were generated using Origin 2019b (Origin Lab Co., Northampton, MA, USA) and significant difference analysis was carried out on data using SPSS 23.0 (IBM SPSS Inc., Chicago, IL, USA). All data are presented as mean values, with standard deviations represented by error bars.

## 3. Results and Discussion

### 3.1. Morphological Characterization of Nano-ZnO

Among all the methods for preparing ZnO materials, the solution method is especially simple and environmentally friendly, and it can be used at low temperatures. The application of different levels of EG content is one of the main methods used to prepare ZnO nanomaterials with different structures. Saito et al. [34] discovered that EG has the potential to hinder the growth of ZnO crystals by acting as a capping agent. EG can also facilitate the aggregation of small granular crystals into rounded particles. However, in cases where the concentration of EG/H_2_O is low, these rounded particles disintegrate into individual particles. Because of the high hydrophilicity of EG, the hydrothermal reaction process can be optimized by adjusting the ratio of EG to deionized water.

**Figure 1:**
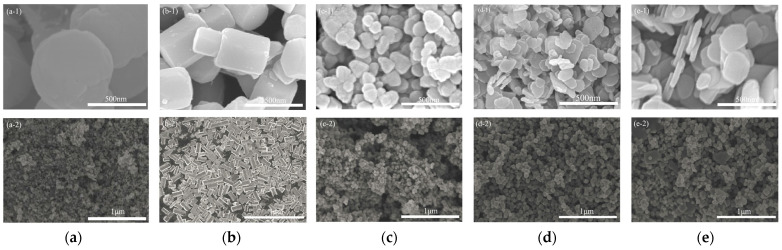
SEM images of ZnO nanomaterials. (**a**) S-ZnO; (**b**) C-ZnO; (**c**) G-ZnO; (**d**) F-A-ZnO; (**e**) F-B-ZnO. (**a-1**,**a-2**) are different enlarged sizes of the same material.

In the present study, the surface morphology of the ZnO material was observed by SEM as illustrated in Figure 1. When the ratio of EG to H_2_O is 2: 1, nanospheres with a diameter of about 500–600 nm are obtained; these are termed spherical ZnO (S-ZnO). There are depressions at both ends of the spherical particles and folds in the depressions, as shown in Figure 1a. When the ratio of EG to H_2_O is adjusted to 1: 1, cylindrical particles with a length of about 600–800 nm are obtained (Figure 1b); these are termed cylindrical ZnO (C-ZnO). Cylindrical structures may be tightly bound as a result of interactions between small particles. Figure 1c shows that, when the EG/H_2_O ratio is 1:3, the granular ZnO (G-ZnO) is composed of irregular ZnO particles with diameters of 50–150 nm. Due to the intermolecular force, the particles are closely bound to each other. When the EG/H_2_O ratio is adjusted to 1: 4, ZnO nano-flakes with diameters of approximately 150–300 nm and thicknesses of approximately 30 nm are obtained (Figure 1d. Due to electrostatic or intermolecular forces, the flake structures are piled up; this is termed flake (A) ZnO (F-A-ZnO). However, when the EG/H_2_O ratio is adjusted to 1: 6, ZnO nano-flakes with a similar morphology to F-A-ZnO but with a larger flake diameter (about 200–400 nm) are obtained; this is termed flake (B) ZnO (F-B-ZnO), as shown in Figure 1e. The findings indicate that the morphology of the prepared ZnO is significantly influenced by the ratio of EG to H_2_O in the solution used for the synthesis of nano-ZnO.

### 3.2. Comparison of Performance of ZnO@Lac with Different Morphologies

In this experiment, the conditions of immobilizing laccase with ZnO with five different morphologies were optimized by an orthogonal experiment. Temperature, adsorption time, and enzyme concentration were all controlled, with temperatures of 20, 30, 40, 50, and 60 °C, adsorption times of 0.5, 1, 2, 3, and 4 h, and enzyme concentrations of 5, 10, 15, 20, and 25 mg/mL. Significance analysis was conducted using SPSS software; the results are shown in Table 1. Under conditions of 30 °C, 0.5 h, and 25 mg/mL, G-ZnO@Lac exhibited the highest recovery, of 48.35%, with the load capacity being 1058.42 mg/mL. S-ZnO@Lac had a higher load capacity of 1258.31 mg/mL, but recovery was only 23.98%. Given that the activity of the enzyme represents the most crucial factor in the degradation of MES, we concluded that the best immobilization carrier was G-ZnO.

### 3.3. Characterization of Free Laccase and Immobilized Laccase

**Figure 2:**
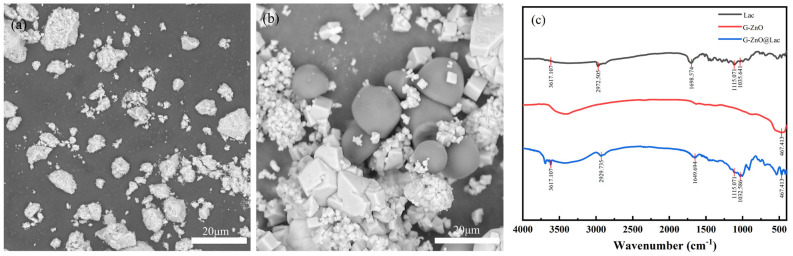
SEM images of granular ZnO (**a**) and ZnO@Lac (**b**); FTIR spectra of laccase, G-ZnO, and G-ZnO@Lac (**c**).

To observe the morphologies of free laccase and immobilized laccase, the immobilized laccase prepared under the best immobilization conditions was chosen. Figure 2a, b show an SEM scan image of G-ZnO material and G-ZnO@Lac. It can be seen that the G-ZnO material exhibits an irregular square shape under low magnification, with G-ZnO@Lac being similar to the main morphology of the G-ZnO material. The white part is made of the ZnO material, and the dark spherical part is made of laccase. It can also be observed that laccase binds well to the granular ZnO material. These results demonstrate that laccase can be well adsorbed on G-ZnO materials through adsorption.

Figure 2c is a Fourier infrared spectrogram (FT-IR) of laccase, granular ZnO (G-ZnO), and granular ZnO-immobilized laccase (G-ZnO@Lac). The 470 cm^−1^ frequency is indicative of the vibration of the Zn–O bond in granular zinc oxide [35]. The peak at around 1035 cm^−1^ is indicative of the stretching vibration of C-N found in laccase, and the absorption peak at 1115 cm^−1^ may be attributed to the stretching vibration of C-O [36]. The vibration of asymmetric and symmetric stretching of the C-H bands of laccase is represented by the bands at 2972 cm^−1^. The vibration at 1698 cm^−1^ may be ascribed to the C=C bonds [37]. Moreover, the presence of a peak at 3617 cm^−1^ in G-ZnO@Lac suggests the presence of an amide bond in the laccase protein, thereby confirming the immobilization of laccase on the G-ZnO surface [38].

### 3.4. Construction of Immobilized Laccase System

#### 3.4.1. pH and Temperature Stability of Laccase and Immobilized Laccase

**Figure 3:**
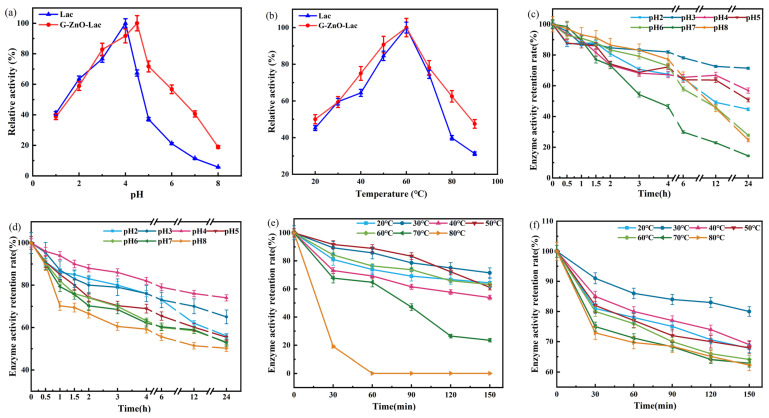
Effects of pH (**a**) and temperature (**b**) on the activities of laccase and G-ZnO@Lac; pH stability of laccase (**c**) and G-ZnO@Lac (**d**); and thermal stability of laccase (**e**) and G-ZnO@Lac (**f**).

Under different conditions (pH, temperature), the activity of laccase before and after immobilization was measured with ABTS as substrate. The pH stability of laccase stored for one day at pH 2–8 and the temperature stability of laccase stored for 2.5 h at 20–80 °C were compared. Figure 3b illustrates that the optimal temperature for free laccase and immobilized laccase is 60 °C. However, the optimal pH values (Figure 3a) are different for free laccase and immobilized laccase, being pH 4 and pH 4.5, respectively. After storage for one day, the enzyme activity retention rates of free laccase and immobilized laccase were 71.00% and 74.00%, respectively (Figure 3c,d). The stability of immobilized laccase in buffer solutions with different pH values is generally higher than that of free laccase, indicating that G-ZnO@Lac is conducive to the enhancement of pH stability of laccase. The observed reduction in enzyme activity at alkaline pH may be attributed to the presence of hydroxide ions, which affect the internal structure of the T2/T3 copper in the laccase molecule. This leads to a reduction in the oxygen reduction potential, thereby hindering the catalytic reaction of laccase and reducing the efficiency of the reaction [36]. Additionally, the enzyme molecules are protected by carriers to avoid denaturation and inactivation caused by harsh reaction conditions. From Figure 3e,f, it may be seen that free laccase is inactivated after being stored at 80 °C for one hour, the enzyme activity retention rate maintains a level above 50.00% between 20 and 60 °C, and the retention rate of immobilized laccase remains above 60.00% between 20 and 80 °C, being 62.30% at 80 °C. We may say, then, that the thermal stability of immobilized laccase is significantly enhanced. The immobilized laccase retains more activity at high temperatures because the conformational fluidity of the immobilized laccase molecule is limited, and this enhances the thermal stability of the laccase [39]. Moreover, increasing the temperature within a certain range also helps to overcome the diffusion resistance between the substrate molecule and the laccase molecule, leading to an increase in the relative activity of the immobilized laccase [40]. Therefore, in comparison to free laccase, the adaptive range of immobilized laccase to pH and temperature is improved, and the stability of pH and temperature is enhanced.

#### 3.4.2. Storage Stability of Laccase and Immobilized Laccase

**Figure 4:**
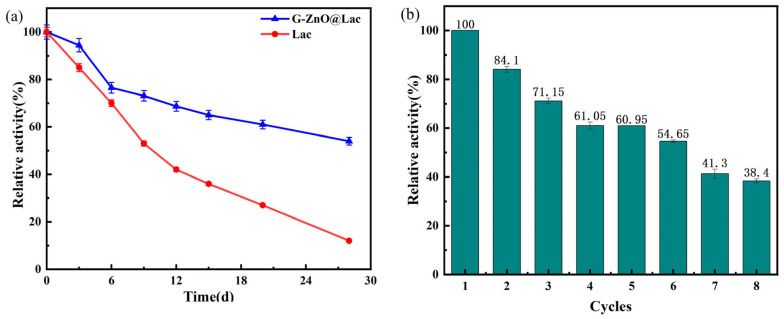
Storage stability of laccase (**a**); and reusability of immobilized laccase (**b**).

Laccase and G-ZnO@Lac were stored at 4 °C for 28 days. Enzyme activity testing was conducted at 0, 3, 6, 9, 12, 15, 20, and 28 d, and the results are shown in Figure 4a. It can be seen that both laccase and G-ZnO@Lac exhibit a continuous and stable downward trend over the 28 days, but the downward trend of G-ZnO@Lac is relatively slow. In addition, the enzyme activity retention rate of G-ZnO@Lac is higher than that of laccase, indicating that immobilized laccase has significantly enhanced storage stability compared with free laccase. The ZnO carrier protects the structure of laccase and prevents the loss of enzyme activity caused by molecular agglomeration during the use and recycling of laccase, thus making it more stable [39,41].

#### 3.4.3. Reusability of Immobilized Laccase

At the optimal reaction temperature and pH value, immobilized laccase was used in eight consecutive reaction cycles. The results are presented in Figure 4b. After five cycles, the relative enzyme activity was still as high as 60.95%. After eight cycles, the relative enzyme activity still exceeded the original 38.40%, indicating good reusability. One possible cause for this loss of enzyme activity could be the immobilization of laccase through adsorption as the binding force between the carrier and laccase is poor. Washing during the operation reaction can easily lead to partial denaturation and deactivation of the immobilized laccase, as well as a small amount of laccase falling off in the pores of the carrier [42].

#### 3.4.4. Kinetic Parameters

**Figure 5:**
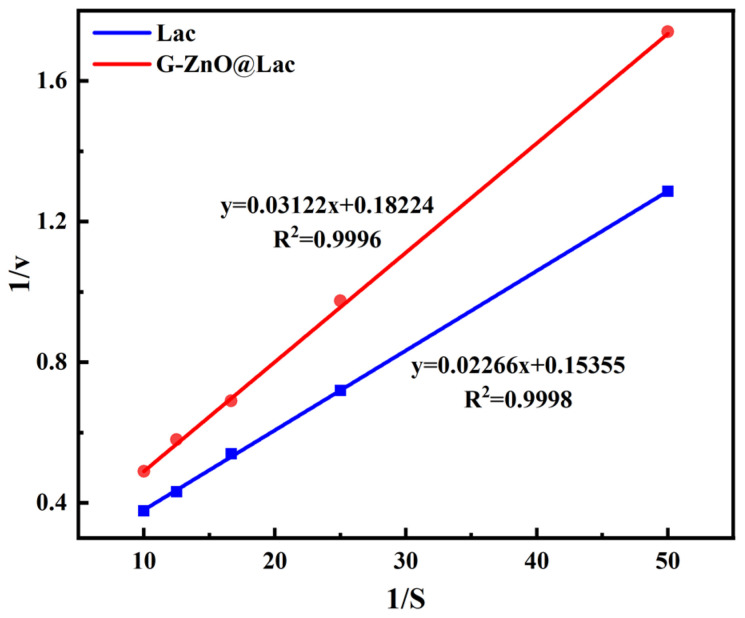
The affinity of laccase and G-ZnO@Lac to the substrate.

The kinetics of the enzymatic reaction between free laccase and immobilized laccase were discussed. The results are shown in Figure 5. The observed slight decrease in V_max_ for immobilized laccase (0.55 mM/min) compared with free laccase (0.65 mM/min), could have resulted from the constrained mobility and decreased availability of active sites in the immobilized enzyme [38]. This reduction should not be related to differences in affinity following immobilization, as the values of K_m_ were similar before and after immobilization (free laccase: 0.15mM; immobilized laccase: 0.17mM).

Such variation in kinetic parameters has been observed in other studies. For example, Dos Santos et al. [43] co-immobilized laccase on agarose-based supports and found a similar trend to that identified in the present work; the V_max_ values of the laccase showed a slight decrease upon immobilization (around 0.13 mM/min), but the values of K_m_ did not significantly change (around 0.04 mM). Ruqayah et al. [38] immobilized laccase on modified multiwall carbon nanotubes. They found that the variation in K_m_ values was insignificant, while V_max_ values decreased. These results indicate that kinetic parameters are affected by the nature of the carrier and by the type of immobilization. However, because of the specific conditions of the activity assays (e.g., pH), an accurate comparison of our results with those reported in the literature is often difficult [44].

### 3.5. Optimization of Degradation Conditions of MES by Immobilized Laccase

**Figure 6:**
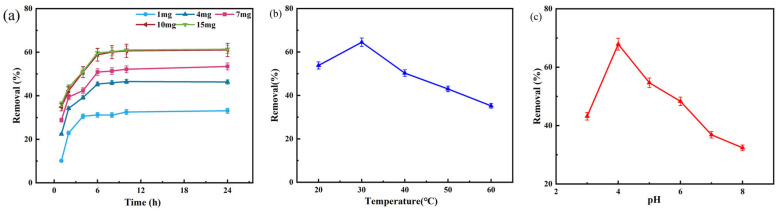
Effects of the following factors on the degradation rate of MES: initial dosage of immobilized laccase and reaction time (**a**); temperature (**b**); and pH (**c**).

The effects of the factors of reaction time, initial dosage of G-ZnO@Lac, temperature, and pH on the degradation of MES by immobilized laccase were investigated. Figure 6a illustrates the impact of reaction time (0–24 h) and initial dosage of G-ZnO@Lac (1 mg, 4 mg, 7 mg, 10 mg, and 15 mg) on the removal of the target. An increased concentration of G-ZnO@Lac was found to promote the removal of contaminants to a certain degree due to an increase in immobilized laccase providing more active enzyme sites. However, the concentration of immobilized laccase increased continuously, resulting in an increase in ZnO material, resulting in an agglomeration phenomenon, which hindered the binding of MES to the active site of laccase; on the other hand, the contact with the carrier covered the active center of laccase, resulting in a lower degradation rate [45]. Considering the economic factors and the degradation rate, a 10 mg dosage of immobilized laccase was used under the conditions of this experiment, and the degradation rate of immobilized laccase on MES was found to reach 59.78% after 6 h.

As can be seen in Figure 6b, the removal reached a maximum level of 64.5% at 30 °C. The removal rate of MES exhibited a trend of initial increase followed by a subsequent decline, because within a certain temperature range, the higher the temperature, the higher the enzyme activity. However, the dissolved oxygen concentration in the reaction system decreases when the temperature is too high, and the lower level is not enough to support the higher reaction rate at high temperatures, leading to a decrease in removal [41,46]. It is apparent from Figure 6c that the optimal pH value of immobilized laccase for degradation of MES was 4, and the degradation rate was 67.90%. Hence, the best conditions for degradation of MES by G-ZnO@Lac were determined to be 10 mg, 6 h, 30 °C, and pH 4. Under these conditions, the degradation efficiency of immobilized laccase on MES can reach 73.25%.

### 3.6. Analysis of the Degradation Mechanism of Mesotrione

**Figure 7:**
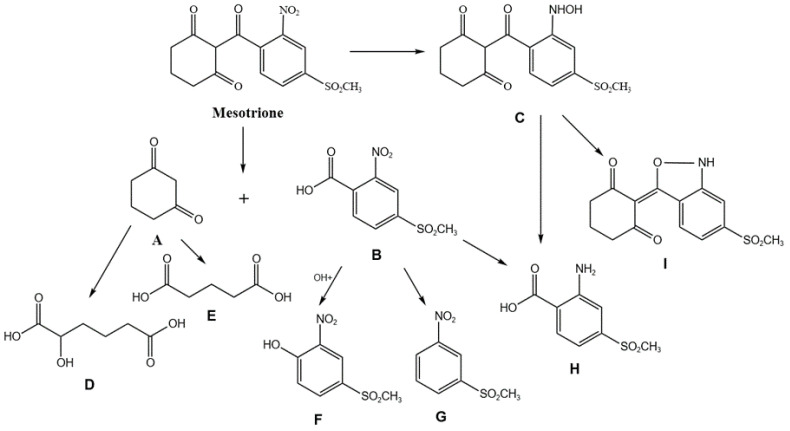
There may be several degradation products from MES.

Mesotrione, (2-(4-methanesulfonyl-2-nitrobenzoyl) cyclohexane-1,3-dione, C_14_H_13_NO_7_S, 36 atoms, MES) is a triketone herbicide with different functional groups attached to the benzene ring. Depending on the catalytic oxidation of laccase, and on the molecular structure and synthetic pathway of MES, there may be several degradation products in the degradation process of MES by laccase. After oxidation and fragmentation of MES molecules, cyclohexane-1,3-dione (CHD, A), and 4-(methylsulfonyl)-2-nitrobenzoic acid (MNBA, B) were produced. The mesotrione hydroxylamine derivative (C) is formed due to reduction in the nitro fraction of MES. In addition, 2-hydroxyhexanedioic acid (D) and pentanedioic acid (E) are generated by further fragmentation and hydroxylation of CHD (A); MNBA is an intermediate product of oxidation and does not accumulate in the degradation process. Hydroxyl substitutes carboxyl in MNBA, resulting in 1-nitro-3-methylsulfonylphenol (F) and 1-(methylsulfonyl)-3-nitrobenzene (G) products; product C is further catalytically degraded to produce 2,1-benzisoxazole-like mesotrione derivative (I). Moreover, 4-(methylsulfonyl)-2-aminobenzoic acid (AMBA) (H) is formed by the reductive transformation of the NO_2_ moiety through the formation of intermediates bearing NO and NHOH groups [4,47,48,49,50]. The degradation pathways are shown in Figure 7.

## 4. Conclusions

In summary, in the present study, we prepared the immobilized laccase G-ZnO@Lac using granular ZnO as a carrier. The optimum immobilization conditions were found to be 30 °C, 0.5 h, and 25 mg/mL. The enzyme activity recovery and immobilization capacity were found to be 48.35% and 1058.42 mg/mL, respectively. The physical protections of G-ZnO on laccase resulted in enhanced thermal stability, acid–base stability, storability, and reusability. Moreover, G-ZnO@Lac maintained high relative activity at temperatures of 20–80 °C with pH values of 2–6, and this represented an improvement compared with free laccase. The relative activity of immobilized laccase remained above 50% (free laccase: 12%) after 28 days of storage. On this basis, the optimal degradation conditions of G-ZnO@Lac for MES were investigated. Under optimal conditions (10 mg, 6 h, 30 °C, and pH 4), the degradation efficiency reached 73.25%. This study provides a theoretical basis for the application of immobilized laccase in the field of herbicides.

## Figures and Tables

**Table 1:** Optimizing conditions for immobilization of laccase with different morphologies of ZnO.

Support Material	S-ZnO	C-ZnO	G-ZnO	F-A-ZnO	F-B-ZnO
Enzyme activity recovery rate (%)	23.98	27.54	48.35	25.12	35.73
Fixed load capacity (mg/mL)	1258.31	1153.27	1058.42	932.48	830.21

## Data Availability

The data presented in this study are available on request from the corresponding author.
